# A nationwide population-based study to access the risk of metachronous esophageal cancers in head and neck cancer survivors

**DOI:** 10.1038/s41598-020-57630-6

**Published:** 2020-01-21

**Authors:** Chao-Ming Tseng, Hsi-Hao Wang, Ching-Tai Lee, Chi-Ming Tai, Cheng-Hao Tseng, Chih-Cheng Chen, Ying-Nan Tsai, Tzu-Haw Chen, Ming-Hung Hsu, Chih-Chun Wang, Tzer-Zen Hwang, Hsiu-Po Wang, Wen-Lun Wang

**Affiliations:** 1 Department of Internal Medicine, E-Da Hospital/I-Shou University, Kaohsiung, Taiwan; 2 Department of Internal Medicine, E-Da Cancer Hospital, Kaohsiung, Taiwan; 3 Department of Otolaryngology, E-Da Hospital/I-Shou University, Kaohsiung, Taiwan; 4 Department of Internal Medicine, National Taiwan University Hospital, Taipei, Taiwan

**Keywords:** Gastroenterology, Oesophagogastroscopy

## Abstract

How long esophageal screening should be performed for, and on which sub-groups of head and neck cancer (HNC) survivors, remains uncertain. This retrospective study analyzed data from the Taiwan National Health Insurance Research Database from 1999 to 2013. A total of 68,131 newly- diagnosed HNC patients were enrolled. Subjects who received esophageal endoscopic screening within 6 months after their diagnosis date of index HNC were identified. The incidence trends of secondary primary EC were analyzed using a Cochran-Armitage trend test. Among the 9,707 patients who received index esophageal endoscopy screening, 101 (1.0%) cases of synchronous EC were diagnosed. The 5- and 10-year cumulative incidence rates of metachronous ECs were 1.4% and 2.7%, respectively in those with an initial negative index endoscopic finding. Patients with oropharynx or hypopharynx cancers were at significantly higher risk of developing metachronous ECs compared with those with oral or larynx cancers (10-year incidence rate: 3.3% *vs*. 0.9%, respectively; hazard ratio: 2.15; 95% confidence intervals: 1.57–2.96). Metachronous EC continues to develop in patients with HNC even at 10-years after treatment for primary HNC. HNC patients, especially those with oropharynx or hypopharynx cancer, may require long-term endoscopic surveillance.

## Introduction

Head and neck cancers (HNC), including oral cavity, oropharynx, hypopharynx and larynx cancers, are major health threats worldwide; in 2015 there were approximately 809,000 new cases and approximately 316,000 deaths due to HNC^1^. As explained by the ‘field cancerization’ theory, patients with HNC tend to have secondary primary tumors^2^. The head and neck, the lungs and the esophagus were the most commonly involved sites^3,4^. The incidence of secondary primary tumors has been reported to vary from 14 to 36%^3,5,6^. A worse prognosis was reported in HNC patients with secondary primary tumors^4,7^.

Esophageal cancer (EC) was one of the most common types of secondary primary tumor, with standardized incidence ratios of 7.2 within the first year of diagnosis with primary HNCs^3^. These ratios remained high even after 10 years of diagnosis with primary cancer^3^. The risk of secondary EC differed significantly according to the subsite of HNC; hypopharyngeal cancer and orpharyngeal cancer were most commonly associated with the occurrence of secondary EC^8^. Endoscopic surveillance is recommended for patients with HNC^9^. Compared with the non-routine screening group, the screening group tended to detect more patients with EC during the early stages^10^. In addition, image-enhanced endoscopy was reported to be a useful tool for risk stratification and prognosis prediction^11^. To date, there have been few large cohort studies to explore the incidence of metachronous EC^4^, and how long EC screening should be performed in HNC survivors remains unclear. The current population-based study with long-term follow-up aimed to examine the incidence of synchronous and metachronous EC after diagnosis with HNC, and to stratify the necessity of endoscopic surveillance.

## Materials and Methods

### Data sources

The present nationwide population-based study examined data on HNC from the National Health Insurance Research Database (NHIRD) in Taiwan. The NHIRD is the administrative database for the Taiwan Health Insurance (NHI) program, and it covers the health care of >99% of the population. The completeness and accuracy of the NHIRD are guaranteed by the Department of Health and the NHI Bureau of Taiwan. The insurance authority releases the insured medical records to the public as de-identified secondary data for the purpose of research. The Registry for Catastrophic Illness Patient Database (RCIPD) is a subset of the NHIRD. It is a registry system for catastrophic disease, including end-stage renal disease, cirrhosis, autoimmune disease, congenital abnormalities and cancers. Entry into this registry can exempt individuals from copayment, and therefore requires explicit criteria for entry, such as imaging or pathological diagnosis^12^. The quality of Catastrophic Illness Patient Database is guaranteed by the Taiwan Health Insurance Bureau where a peer review of pathology and imaging is conducted. The authors requested the RCIPD from the institute, and all included individuals were followed up until the end of 2013 for outcome identification using the International Classification of Disease, 9th Revision, Clinical Modification (ICD-9-CM). All methods were carried out in accordance with relevant guidelines and regulations. Informed consents were waived due to analysis of national database. Data acquisition and analysis were approved by the institutional review board of the E-Da Hospital (EMRP-107-066).

### Study participants and outcomes

From 1999-2012, all newly diagnosed cases of HNC were extracted from the RCIPD, based on their ICD-9-CM criteria (oral cavity: ICD-9-141, 143–145; oropharynx: ICD-9-146; hypopharynx: ICD-9-148; larynx: ICD-9-161). The index date for all HNC patients was assigned as their date of diagnosis with HNC. Patients were excluded if they had any other cancers before the index date or they were less than 20 years old.

The timing of index esophagogastroduodenoscopy (EGD) was identified, and the patients were grouped accordingly into i) those with esophageal endoscopic screening within 6 months after the diagnosis of index HNC; or ii) those without an esophageal endoscopy. Synchronous EC was defined as EC detected within 6 months of the index date. Metachronous EC was defined as the occurrence of newly diagnosed EC 6 months after a negative index EGD. The primary outcome was the cumulative incidence of synchronous and metachronous EC in patients with HNCs.

### Data analysis and statistical methods

Data are presented as the mean ± standard deviation or the number and percentage. Categorical variables were compared using the chi-squared test and continuous variables were analyzed using student’s t-test. Hazard ratios (HRs) and 95% confidence intervals (CIs) were calculated using the Cox proportional hazard model. All statistical tests were two-sided, conducted at a significance level of 0.05, and reported using a P-value and/or 95% CIs. Incidence pattern trends were analyzed using a Cochran-Armitage trend test. All analyses were performed using SAS software, version 9.4 (SAS Institute, Cary, NC, USA).

## Results

### Baseline characteristics of the study participants

**Figure 1:**
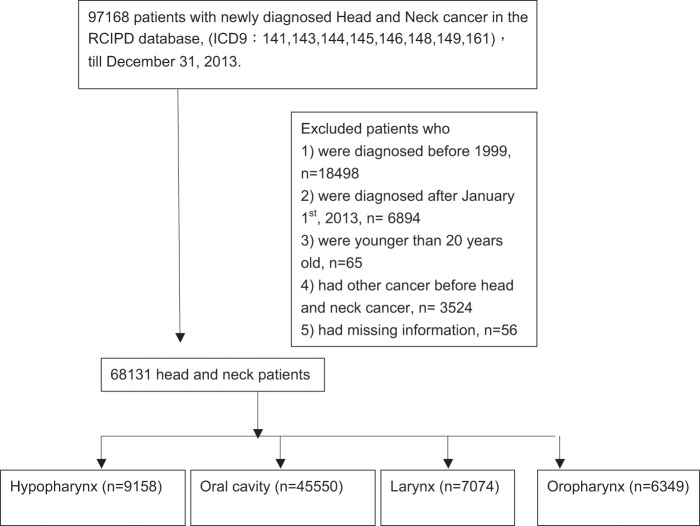
Flow diagram showing the enrollment of patients with head and neck cancers from the database.

A flowchart of the enrollment process for the study cohort is shown in Fig. 1. A total of 97,168 newly diagnosed HNC patients were identified from the RCIPD. Patients who i) were diagnosed before 1999, n = 18,498; ii) were diagnosed after January 1^st^ 2013, n = 6,894; iii) were younger than 20 years old, n = 65; iv) had other cancers before their diagnosis of HNC, n = 3,524; and v) had missing information, n = 56, were excluded. In total, 68,131 HNC patients were enrolled from between 1999 and 2012. These patients were further stratified according to the location of their HNC (oral cavity, n = 45,550; hypopharynx, n = 9,158; oropharynx, n = 6,349; and larynx, n = 7,074).

The demographic characteristics of the enrolled HNC patients that were stratified by tumor location are shown in Table 1. The majority of the patients were male (>90%) and more than 50 years-old (>50%). Except for those with oral cavity cancer, more than 20% of HNC patients received esophageal endoscopic examinations (index EGD) within 6 months of their diagnosis with HNC.

**Table 1:** Baseline characteristics in head and neck cancer patients.

Variable	Oral cavity (n = 45550)	Oropharynx (n = 6349)	Hypopharynx (n = 9158)	Larynx (n = 7074)
Age, years (mean ± SD)	53.49 ± 12.19	54.59 ± 11.38	56.84 ± 11.97	64.35 ± 12.20
20–29	465 (1.0)	24 (0.4)	20 (0.2)	12 (0.2)
30–39	5137 (11.3)	435 (6.9)	478 (5.2)	121 (1.7)
40–49	13681 (30.0)	1953 (30.8)	2440 (26.6)	827 (11.7)
50–59	13846 (30.4)	2144 (33.8)	2922 (31.9)	1683 (23.8)
60–69	7539 (16.5)	1117 (17.6)	1840 (20.1)	1887 (26.7)
70+	4873 (10.7)	676 (10.7)	1458 (15.9)	2544 (36.0)
Male	41218 (90.5)	5760 (90.7)	8804 (96.1)	6676 (94.4)
Index EGD	5272 (11.6)	1383 (21.8)	3988 (43.6)	1415 (20.0)

EGD: esophagogastroduodenoscopy.

A total of 9,707 patients were identified as receiving index EGD screening (Table 2). The percentage of patients who received early endoscopic evaluation increased gradually, from 8.6% in 1999 to 22.2% in 2012. Among them, 101 patients were confirmed as having been diagnosed with EC within 6 months of their index HNC diagnosis date.

**Table 2:** Clinical characteristics in patients receiving early endoscopic evaluation.

Variable	Endoscopic screening (n = 9707)
**Age**, years (mean ± SD)	56.05 ± 12.19
20–29	38 (0.4)
30–39	730 (7.5)
40–49	2518 (25.9)
50–59	2984 (30.7)
60–69	2025 (20.9)
70 +	1412 (14.5)
**Male**	9113 (93.9)
Year, number (%*)	
1999	283 (8.6)
2000	307 (8.9)
2001	361 (9.9)
2002	394 (10.0)
2003	458 (10.7)
2004	539 (11.7)
2005	565 (12.2)
2006	592 (11.6)
2007	664 (12.3)
2008	735 (13.4)
2009	976 (16.4)
2010	1125 (18.7)
2011	1326 (21.6)
2012	1382 (22.2)
**Tumor location**	
Oral cavity	5157 (53.1)
Oropharynx	1108 (11.4)
Hypopharynx	2175 (22.4)
Larynx	1267 (13.1)

*Patients receiving early endoscopic evaluation/Patients with newly diagnosed head and neck cancer each year.

### Incidence and trends of second primary EC

**Figure 2:**
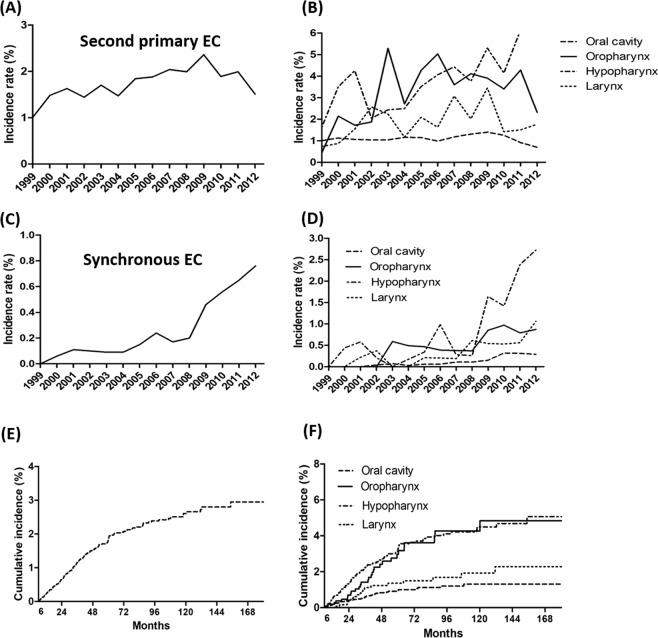
(**A**) The trend of incidence of second primary esophageal cancer by year; (**B**) The trend of second primary esophageal cancers, stratified by head and neck cancer location; (**C**) The trend of incidence of synchronous esophageal cancer by year; (**D**) The trend of synchronous esophageal cancer, stratified by head and neck cancer location; (**E**) The cumulative incidence of metachronous esophageal cancer in patients with negative index endoscopic screening finding; (**F**) The cumulative incidence of metachronous esophageal cancer, stratified by head and neck cancer location.

The cumulative incidence rate of second primary EC in HNC patients increased significantly between 1999 and 2009, from 1% in 1999 to 2.4% in 2009 (Fig. 2A). This significant increase in the incidence rate remained even after stratification by HNC tumor location (hypopharynx: from 1.6% in 1999 to 5.3% in 2009, *p* = 0.001; oropharynx: from 0.4% in 1999 to 3.9% in 2009, p = 0.002; larynx: from 0.7% in 1999 to 3.4% in 2009, *p* = 0.002), except for oral cavity cancer (from 1.0% in 1999 to 1.4% in 2009, p = 0.100; Fig. 2B). Due to the relatively short follow-up period in patients enrolled between 2010 and 2012, the cumulative incidence of second primary EC was lower.

Figure 2C shows that the annual incidence rate of synchronous EC increased significantly during the study period. This increasing trend remained after stratification by each HNC tumor location (hypopharynx: from 0% in 1999 to 2.72% in 2012, *p* < 0.001; oropharynx: from 0% in 1999 to 0.87% in 2012, *p* = 0.003; larynx: from 0% in 1999 to 1.06% in 2012, *p* < 0.001; oral cavity: from 0% in 1999 to 0.29% in 2012, *p* < 0.001; Fig. 2D).

### Cumulative incidence of metachronous EC

Figure 2E shows the cumulative incidence of metachronous EC in patients with a negative finding at their index EGD screening. The 5- and 10-year cumulative incidence rates of metachronous EC were 1.4% and 2.7%, respectively. This cumulative incidence in the metachronous EC rate continued to rise even after the 10-year follow-up period. The incidence of metachronous EC stratified by HNC location is shown in Fig. 2F. Patients with hypopharynx or oropharynx cancers had a higher metachronous recurrence risk compared with those with larynx or oral cavity cancer. The high-risk patients (hypopharynx or oropharynx cancers) had significantly higher 5- and 10-year cumulative incidence rates of metachronous ECs compared with the low-risk patients (larynx or oral cavity cancer). The 5-year cumulative incidence rates of the high- and low-risk patients were 2.7% *vs*. 0.8% (HR: 2.35; 95% CI:1.66–3.32), respectively and the 10-year cumulative incidence rates were 3.3% *vs*. 0.9% (HR: 2.15; 95% CI:1.57–2.96), respectively (Table 3). Supplement Fig. 1 shows the cumulative incidence of metachronous EC in patients not receiving index EGD screening. Patients with hypopharynx or oropharynx cancers also had a higher metachronous recurrence risk. The 5-year cumulative incidence rates of the high- and low-risk patients were 2.8% *vs*. 0.8% (HR: 3.90; 95% CI:3.37–4.53), respectively and the 10-year cumulative incidence rates were 3.4% *vs*. 1.1% (HR: 3.57; 95% CI:3.14–4.07), respectively (Supplement Table 5).

**Table 3:** Risk of metachronous esophageal cancer between low and high-risk patients.

	Low risk (n = 6371)	High risk (n = 3235)	HR (95% CI)
5-yrs Case No (%)	51 (0.8)	87 (2.7)	2.35 (1.66, 3.32)
10-yrs Case No (%)	59 (0.9)	107 (3.3)	2.15 (1.57, 2.96)

## Discussion

Due to recent advances in the diagnosis and treatment of HNC, an increasing number of patients may survive more than 5–10 years. Therefore, metachronous EC could become an important public health issue in the future. However, to date no studies have investigated how long HNC patients are at an increased risk of developing metachronous esophageal neoplasia. To the best of our knowledge, the present population-based long-term follow-up study is the first to report that metachronous esophageal neoplasia may continue to develop even 10 years after HNC treatment. Although the awareness of endoscopic screening for EC in HNC patients has improved over recent years, the results of the current study may guide endoscopic surveillance policy. The findings suggest that esophageal screening should be performed for a minimum of 10 years, especially in patients with oropharynx or hypopharynx cancer.

As explained by the field cancerization theory, patients with HNC frequently develop second primary ECs. Several prospective studies have shown that the prevalence of synchronous EC is 15–20% per year^5,13^, while metachronous cancer is approximately 2% per year^5^. If they are not detected via esophageal screening, these concomitant esophageal tumors can become symptomatic, and even metastasize, which can result in a poor prognosis. The authors previously reported the benefits of esophageal screening in patients with HNC; it led to an increase in 3-year survival rates from 40% to 60%^14^. This previous study also found that narrow-band imaging with magnification was the best modality for screening^15^. However, how long HNC patients should be screened for, remained unclear. The current study confirmed that HNC patients may develop metachronous ECs, even though the index EGD showed a negative result. Therefore, regular endoscopic follow-ups are recommended and the standard surveillance period should be increased.

The present study demonstrated that the percentage of HNC patients receiving early esophageal screening has gradually increased from 8.6% in 1999 to 22.2% in 2012. This probably represents the effects of awareness campaigns on the risk of synchronous EC for patients with HNC. A previous population-based study in Taiwan included 2,965 subjects who had received their first-time diagnosis of oral/oropharyngeal/hypopharyngeal cancer in 2002–2009; it reported the prevalence of synchronous EC as 2.19%^16^. The present study enrolled all HNC patients (oral/oropharyngeal/hypopharyngeal/larynx) from 1999 to 2012 in Taiwan. A total of 9,707 of them received index esophageal endoscopy screening, and 101 (1.0%) cases of synchronous EC were diagnosed. The difference of synchronous rate between current study and previous study^16^ might be caused by different study design and definitions of outcome. In previous study, synchronous esophageal EC was defined as EC detected within 3 months before and after the index date. Nevertheless, synchronous EC was defined as EC detected after 6 months of the index date in this study and we excluded the patients with cancers before the diagnosis of HNC. As demonstrated in Fig. 2C, the incidence of synchronous EC increased significantly during the study period, especially after 2009. Although the incidence rate may be underestimated in a retrospective study, it is expected that with improved awareness, national training programs and new endoscopic modalities, the incidence of secondary EC will increase.

Early detection and prompt treatment of EC are crucial for improving a patients’ chances of survival. It has been reported that half of all detected synchronous cancers are at an early stage, which may be asymptomatic and easily missed via conventional endoscopy^13^. Therefore, some studies recommended the use of routine endoscopic esophageal screening for HNC patients, using an image-enhanced endoscopy^16,17^. The American Society of Clinical Oncology recommends symptom oriented esophageal screening in HNC survivors^18^. Nevertheless, the incidence of synchronous esophageal cancer might differ between eastern and western countries^5,13,19,20^. Compared with Western countries, studies from Eastern Asia have demonstrated a higher incidence of synchronous EC. Alcohol drinking, tobacco smoking and betel nut chewing, as well as the ALDH-2 polymorphism might explain these geographic differences^21,22^. Furthermore, increased awareness of the risk of synchronous EC in patients with HNC may change a physicians’ and local hospital’s treatment guidelines, by using routine endoscopic screening instead of symptom oriented evaluation^10,11,14,20^. Alcohol drinking and tumor location over the oropharynx and hypopharynx, were previously identified by the authors as risk factors for developing synchronous EC. The current study also confirmed that tumor location over the oropharynx and hypopharynx are risk factors for metachronous cancers. In 2011 the Taiwan National Health Research Institute revised its treatment guidelines and recommended the use of endoscopic evaluation in HNC patients, the French Society of Otorhinolaryngology guidelines also recommend systematic exploration as part of pretreatment in at risk patients^23^.

Few studies have addressed the issue of long-term risk for the development of metachronous EC in HNC patients. Su *et al*. demonstrated that metachronous EC could be identified using esophagoscopy in 5.1% of 293 patients with HNC, over a 1-year study period in Taiwan^24^. Another retrospective study of 714 patients in Korea, revealed that 11 (1.5%) patients with metachronous ECs were identified over a median of 31 months^25^. In a study by Petit *el al*. routine esophageal endoscopy screening of patients with treated HNCs was reported to detect metachronous EC in 3.2% of the 1,560 patients in France over a 10-year period^26^. The present study enabled the analysis of the risk of metachronous EC in patients with HNC on a nationwide scale, which differed from previous studies. In our study, patients with hypopharyngeal and oropharyngeal cancer had even higher EC incidence rates over the 10-year follow-up period. These high-risk patients might benefit from long-term endoscopic evaluation. Further prospective studies are warranted to evaluate the survival benefit and cost-effective analysis for screening these high-risk patients.

The current study had several limitations. Firstly, it was a retrospective study, and thus the incidence of second primary EC has most likely been underestimated. Secondly, the study enrolled patients based on their ICD-9 code. The database did not record the histological type of the cancers. Therefore, enrollment of patients with adenocarcinoma was inevitable. Nevertheless, nearly 90% of the EC and HNCs are squamous cell carcinoma in Taiwan^27^, and the conclusions remain the same even after exclusion of patients with non-squamous cell cancers. Thirdly, patients with EC *in situ* or high- grade squamous intraepithelial neoplasia, were not recorded in the RCIPD. This could also lead to under-estimation of the incidence of synchronous and metachronous EC. Fourth, information about substance use and tumor stage were lacking in the database. Sensitivity test was done to investigate the effect of substance use by using alcohol- related disease (ICD-9-CM code 291.xx, 303.xx, 305.0, 571.x, x = 0–3) and chronic obstructive pulmonary disease (ICD-9-CM code 490–496, a surrogate for smoking) (Supplement Tables 1–4). The results remained consistent after stratification. Further prospective studies are warranted to identify and validate the risk factors for developing metachronous ECs. Fifth, the index date of HNC in the RCIPD is actually the date of application instead of date of cancer diagnosis. Previous study reported a delay of median of 15 days after cancer diagnosis was noted in the date of application in the NHIRD^28^. Nevertheless, in this study, we focused on the long- term outcome (more than 10 years). The impact of delayed enrollment on the cumulative incidence rate of metachronous esophageal cancer was expected to be minimal.

## Supplementary information

Supplement information

## Data Availability

We declare that all the data is available.

## References

[1] Fitzmaurice C (2017). Global, Regional, and National Cancer Incidence, Mortality, Years of Life Lost, Years Lived With Disability, and Disability-Adjusted Life-years for 32 Cancer Groups, 1990 to 2015: A Systematic Analysis for the Global Burden of Disease Study. JAMA oncology.

[2] Slaughter DP, Southwick HW, Smejkal W (1953). Field cancerization in oral stratified squamous epithelium; clinical implications of multicentric origin. Cancer.

[3] Chuang SC (2008). Risk of second primary cancer among patients with head and neck cancers: A pooled analysis of 13 cancer registries. International journal of cancer.

[4] Jung YS, Lim J, Jung KW, Ryu J, Won YJ (2015). Metachronous Second Primary Malignancies after Head and Neck Cancer in a Korean Cohort (1993-2010). PloS one.

[5] Muto M (2002). Association of multiple Lugol-voiding lesions with synchronous and metachronous esophageal squamous cell carcinoma in patients with head and neck cancer. Gastrointestinal endoscopy.

[6] Schwartz LH (1994). Synchronous and metachronous head and neck carcinomas. Cancer.

[7] Leon X (1999). Second neoplasm in patients with head and neck cancer. Head & neck.

[8] Morris LG, Sikora AG, Patel SG, Hayes RB, Ganly I (2011). Second primary cancers after an index head and neck cancer: subsite-specific trends in the era of human papillomavirus-associated oropharyngeal cancer. Journal of clinical oncology: official journal of the American Society of Clinical Oncology.

[9] Cohen EE (2016). American Cancer Society Head and Neck Cancer Survivorship Care Guideline. CA: a cancer journal for clinicians.

[10] Su YY (2013). Effect of routine esophageal screening in patients with head and neck cancer. JAMA otolaryngology–head & neck surgery.

[11] Chung CS (2016). Long Term Outcome of Routine Image-enhanced Endoscopy in Newly Diagnosed Head and Neck Cancer: a Prospective Study of 145 Patients. Scientific reports.

[12] Wu CY (2016). Association between ultrasonography screening and mortality in patients with hepatocellular carcinoma: a nationwide cohort study. Gut.

[13] Wang WL (2011). Risk factors for developing synchronous esophageal neoplasia in patients with head and neck cancer. Head & neck.

[14] Wang WL (2013). The benefit of pretreatment esophageal screening with image-enhanced endoscopy on the survival of patients with hypopharyngeal cancer. Oral oncology.

[15] Lee CT (2010). Narrow-band imaging with magnifying endoscopy for the screening of esophageal cancer in patients with primary head and neck cancers. Endoscopy.

[16] Hung SH, Tsai MC, Liu TC, Lin HC, Chung SD (2013). Routine endoscopy for esophageal cancer is suggestive for patients with oral, oropharyngeal and hypopharyngeal cancer. PloS one.

[17] Chung CS (2016). Image-enhanced endoscopy for detection of second primary neoplasm in patients with esophageal and head and neck cancer: A systematic review and meta-analysis. Head & neck.

[18] Nekhlyudov L (2017). Head and Neck Cancer Survivorship Care Guideline: American Society of Clinical Oncology Clinical Practice Guideline Endorsement of the American Cancer Society Guideline. Journal of clinical oncology: official journal of the American Society of Clinical Oncology.

[19] McGarey PO (2016). Rigid Esophagoscopy for Head and Neck Cancer Staging and the Incidence of Synchronous Esophageal Malignant Neoplasms. JAMA otolaryngology–head & neck surgery.

[20] Wang YK, Chuang YS (2017). Endoscopic screening for synchronous esophageal neoplasia among patients with incident head and neck cancer. Prevalence, risk factors, and outcomes..

[21] Chen PT (2011). Incidence and patterns of second primary malignancies following oral cavity cancers in a prevalent area of betel-nut chewing: a population-based cohort of 26,166 patients in Taiwan. Japanese journal of clinical oncology.

[22] Akhtar S (2013). Areca nut chewing and esophageal squamous-cell carcinoma risk in Asians: a meta-analysis of case-control studies. Cancer causes & control: CCC.

[23] Vergez S (2013). Initial staging of squamous cell carcinoma of the oral cavity, larynx and pharynx (excluding nasopharynx). Part I: Locoregional extension assessment: 2012 SFORL guidelines. European annals of otorhinolaryngology, head and neck diseases.

[24] Su YY, Fang FM, Chuang HC, Luo SD, Chien CY (2010). Detection of metachronous esophageal squamous carcinoma in patients with head and neck cancer with use of transnasal esophagoscopy. Head & neck.

[25] Lim H (2015). Clinical significance of early detection of esophageal cancer in patients with head and neck cancer. Gut and liver.

[26] Petit T (2001). Systematic esophageal endoscopy screening in patients previously treated for head and neck squamous-cell carcinoma. Annals of oncology: official journal of the European Society for Medical Oncology.

[27] Lu CL (2010). Increasing trend of the incidence of esophageal squamous cell carcinoma, but not adenocarcinoma, in Taiwan. Cancer causes & control: CCC.

[28] Kao WH (2018). Validity of cancer diagnosis in the National Health Insurance database compared with the linked National Cancer Registry in Taiwan. Pharmacoepidemiology and drug safety.

