# Supplementary material for: A nationwide population-based study to access the risk of metachronous esophageal cancers in head and neck cancer survivors
Source: Sci Rep. 2020 Jan 21;10:884. doi: 10.1038/s41598-020-57630-6 (PMC6972960; doi:10.1038/s41598-020-57630-6)

A nationwide population-based study to access the risk of metachronous esophageal cancers in head and neck cancer survivors

Chao-Ming Tseng, MD [1,2], Hsi-Hao Wang, MD[1], Ching-Tai Lee, MD[1], Chi-Ming Tai, MD[1], Cheng-Hao Tseng, MD[1,2], Chih-Cheng Chen, MD[1,2], Ying-Nan Tsai, MD[1,2], Tzu-Haw Chen, MD[1], Ming-Hung Hsu, MD[1], Chih-Chun Wang, MD[3], Tzer-Zen Hwang, MD[3], Hsiu-Po Wang, MD[4], Wen-Lun Wang*, MD, PhD[1]

[1]*Department of Internal Medicine, E-Da Hospital/I-Shou University, Kaohsiung, Taiwan;*
[2]*Department of Internal Medicine, E-Da Cancer Hospital, Kaohsiung, Taiwan*
[3]*Department of Otolaryngology, E-Da Hospital/I-Shou University, Kaohsiung, Taiwan*
[4]*Department of Internal Medicine, National Taiwan University Hospital, Taipei, Taiwan;*

Reprint requests and correspondence to:

Wen-Lun Wang, MD, PhD

Department of Internal Medicine, E-Da Hospital/I-Shou University,

No. 1, Yida Road, Jiaosu Village, Yanchao District, Kaohsiung City 82445, Taiwan

Tel: 886-7-6150011 ext. 2981; Fax: 886-7-6150940; E-mail: warrengodr@gmail.com

Supplement table 1. Risk of metachronous esophageal cancer between low and high-risk patients with alcohol related disease

	Low risk (n=555)	High risk (n=441)	HR (95% CI)
5-yrs Case No (%)	10 (1.8)	24 (5.4)	2.70 (1.29, 5.64)
10-yrs Case No (%)	10 (1.8)	26 (5.9)	3.20 (1.54, 6.64)

Supplement table 2. Risk of metachronous esophageal cancer between low and high-risk patients without alcohol related disease

	Low risk (n=5816)	High risk (n=2794)	HR (95% CI)
5-yrs Case No (%)	41 (0.7)	63 (2.3)	2.17 (1.47, 3.22)
10-yrs Case No (%)	49 (0.8)	81 (2.9)	1.95 (1.37, 2.79)

Supplement table 3. Risk of metachronous esophageal cancer between low and high-risk patients with chronic obstructive pulmonary disease

	Low risk (n=665)	High risk (n=439)	HR (95% CI)
5-yrs Case No (%)	0 (0.0)	12 (2.7)	NA
10-yrs Case No (%)	1 (0.2)	16 (3.6)	19.50 (2.59, 147.01)

Supplement table 4. Risk of metachronous esophageal cancer between low and high-risk patients without chronic obstructive pulmonary disease

	Low risk (n=5706)	High risk (n=2796)	HR (95% CI)
5-yrs Case No (%)	51 (0.9)	62 (2.3)	2.07 (1.45, 2.95)
10-yrs Case No (%)	58 (1.0)	91 (3.3)	1.85 (1.33, 2.58)

Supplement table 5. Risk of metachronous esophageal cancer between low and high-risk patients not receiving index EGD screening

	Low risk (n=46200)	High risk (n=12224)	HR (95% CI)
5-yrs Case No (%)	358 (0.8)	340 (2.8)	3.90 (3.37, 4.53)
10-yrs Case No (%)	500 (1.1)	409 (3.4)	3.57 (3.14, 4.07)

Supplement figure 1. The cumulative incidence of metachronous EC in patients not receiving index EGD screening

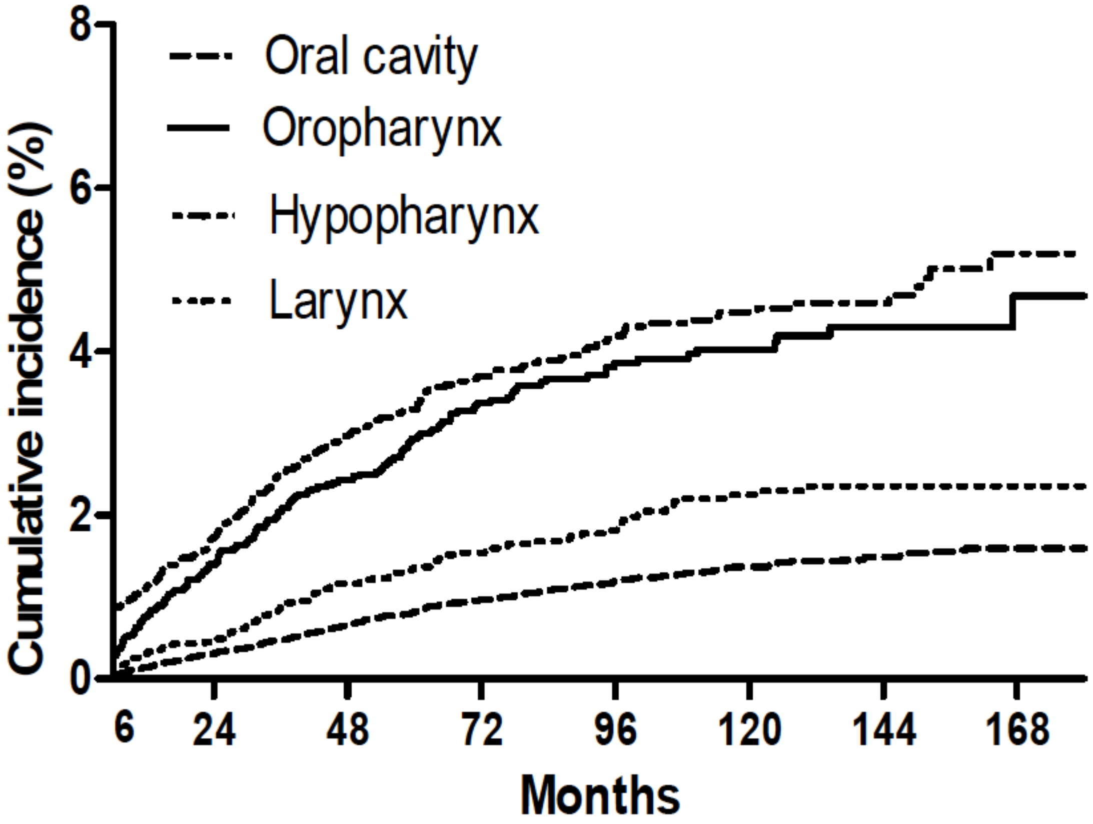

Supplement: Supplementary file 1 — Supplement information [file 41598_2020_57630_MOESM1_ESM.pdf]
